# Metrological Evaluation of Human–Robot Collaborative Environments Based on Optical Motion Capture Systems †

**DOI:** 10.3390/s21113748

**Published:** 2021-05-28

**Authors:** Leticia González, Juan C. Álvarez, Antonio M. López, Diego Álvarez

**Affiliations:** Multisensor Systems and Robotics Group (SiMuR), Department of Electrical, Electronic, Computer and Systems Engineering, University of Oviedo, C/Pedro Puig Adam, 33203 Gijón, Spain; juan@uniovi.es (J.C.Á.); amlopez@uniovi.es (A.M.L.); dalvarez@uniovi.es (D.Á.)

**Keywords:** calibration, groupware, human–robot interaction, industrial robots, optical tracking

## Abstract

In the context of human–robot collaborative shared environments, there has been an increase in the use of optical motion capture (OMC) systems for human motion tracking. The accuracy and precision of OMC technology need to be assessed in order to ensure safe human–robot interactions, but the accuracy specifications provided by manufacturers are easily influenced by various factors affecting the measurements. This article describes a new methodology for the metrological evaluation of a human–robot collaborative environment based on optical motion capture (OMC) systems. Inspired by the ASTM E3064 test guide, and taking advantage of an existing industrial robot in the production cell, the system is evaluated for mean error, error spread, and repeatability. A detailed statistical study of the error distribution across the capture area is carried out, supported by a Mann–Whitney U-test for median comparisons. Based on the results, optimal capture areas for the use of the capture system are suggested. The results of the proposed method show that the metrological characteristics obtained are compatible and comparable in quality to other methods that do not require the intervention of an industrial robot.

## 1. Introduction

Human–robot collaborative shared environments are a topic of growing interest in industry, because of the cost reduction and productivity improvement that come with having robots and humans sharing the same workspace. In this type of production cell, it is mandatory to track the human operator’s behaviour in order to guarantee their safety [1,2,3]. Depending on the task specifications, different sensors can be used to that end, e.g., to measure contact forces, body segment position, or velocity [4].

Optical motion capture (OMC) systems are powerful sensors for human motion tracking. OMCs are widely used in the biomedical fields [5]—for example, biomechanics [6], sports [7], ergonomics, gait analysis [8], gerontology, or rehabilitation [9]. Their accuracy makes them able to be used as a gold standard for evaluating other motion measurement systems [10,11,12]. However, they are also increasingly used in collaborative robots, because a shared workspace equipped with an OMC system can greatly improve the possibility of safe and dynamic interactions between humans and machines [13].

It is necessary to evaluate the accuracy and precision of OMC technology when applied to different human motion scenarios. The rough accuracy specifications provided by manufacturers are easily influenced by various factors affecting measurements, such as (1) calibration procedure (capture volumes/durations) [14], (2) number and resolution of cameras [15], (3) movement condition (static and dynamic), (4) lighting conditions [16], or (5) measurement height (or zone in the captured volume) [17]. Therefore, the individual analysis of the metrological characteristics of an OMC facility is an accepted and recommended practice.

Consequently, different proposals for the assessment of OMC systems have been presented, the main obstacle being the lack of a simple methodology or procedure by which to implement them. A custom-made Cartesian robot was used in [18] to move markers at predefined 30 mm grid points, in order to investigate the influences of system parameters (cameras location, dynamic calibration volume, marker size, and lens filter), but such a system only allows for the covering of small work volumes (0.18 × 0.18 × 0.15 m^3^). A standard test method ASTM E3064 is proposed by the American NIST, based on comparing the relative pose between two sets of markers fixed on a 300-mm-long metrology bar [19,20]. The bar is hand carried at normal walking speed along a test volume as large as 12 × 8 × 2 m^3^. A different protocol is proposed in [21], with emphasis on checking the influence of three factors: the number of cameras, the movement condition, and different heights of interest for biomechanical studies (0, 500, and 1000 mm for foot, knee, and hip, respectively). Errors are all referred to known lengths in the manufacturer’s calibration wand (static) or a two-marker tracking rod (dynamic) to cover a volume of 5.5 × 1.2 × 2 m^3^. A similar static wand length check test is proposed, in order to verify the effect of different calibration procedures on performance [22]. Another proposal to check the absolute accuracy over a 4 × 2.5 × 3 m^3^ volume is through a high-precision 3D scanner used for engineering surveying problems [23]. In [24], the authors used a ThorLabs LTS300 linear motion capture device in order to evaluate the accuracy of an optical system composed of 42 cameras in a capture volume of 135 m^3^. The purpose of [25] was to evaluate the reliability of four different motion analysis laboratories, by applying three simple mechanical tests in order to ensure consistent and comparable data.

This work details the results of a methodology for evaluating the metrological performances of OMC spaces dedicated to human–robot collaboration environments, taking advantage of the availability of industrial robots that can be found in this type of cell. The use of an industrial robot for the evaluation of the calibration process allows the calculation of new statistics, impossible to calculate without it, and creates the possibility of automatizing the assessment of these spaces in a systematic and precise way, avoiding the need to do it manually and without extra instrumentation. Inspired by the ASTM E3064 test guide, the proposed methodology takes the precise movement of the robotic arm as a reference for the calculation of mean error, error spread, and repeatability over the capture area. The resulting errors of the capture system are visualised using a heat map representation, dividing the measurement area into a grid with regular sized cells of 10 × 10 cm^2^. The errors are analysed in detail in order to determine the differences in the performance of the optical system between the boundary zones and the more central zones of the measurement area, studying the overall distribution of the errors and comparing them using a Mann–Whitney U test for median comparisons. Based on the results obtained, the selection of the most optimal measurement area according to the requirements of the experiment is carried out. The system setup, the subsystems calibration, and the complete data analysis and evaluation procedure are described in the following sections.

## 2. Materials and Method

### 2.1. Experimental Setup and Protocol

**Figure 1:**
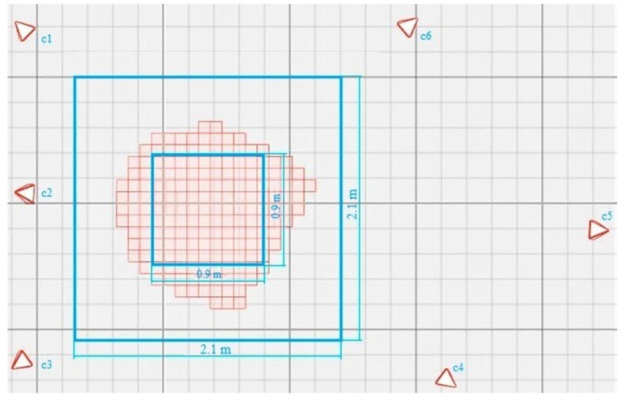
The OMC laboratory setup. The resulting capture areas with an overlap of six cameras (irregular squared red shape), and available workspace (external 2.1 × 2.1 m^2^ blue square).

The optical motion capture system tested consisted of six OptiTrack Flex3 cameras [26] (see Figure 1). The work area to be tested had a measurement volume of approximately 2.1 × 2.1 × 1.5 m^3^. A reduced interior volume of 0.9 × 0.9 × 1 m^3^, which was covered by the six cameras simultaneously, was also analysed. The cameras’ disposition was good enough to overcome the main occlusion problems in our experiments. The results of the interior area will serve to check whether there is any improvement in accuracy as a result of having extra cameras overlapping.

The OMC calibration procedure was performed following the manufacturer’s recommendations, by waving the rigid calibration wand slowly throughout the large empty capture volume, paying special attention to the horizontal plane where the markers would be moved. During the calibration process, the coordinate axis to be used as a reference was located at the centre of the measurement area.

The evaluation procedure consisted of comparing the rigid body pose between two known places, following the basic idea proposed in ASTM E3064 [20]. To move the set of body markers, we used the ABB IRB 120 lightweight manipulator with six rotational joints. A set of four reflective spherical markers (Ø 14 mm) were placed on the object, and its static and dynamic position and orientation were monitored. The robot arm was mounted on a wheeled table with a height of 75 cm in order to move it through the entire measuring area at will. The table was equipped with a braking system that immobilizes the table in order to prevent unwanted movements when the robot arm was in operation.

**Figure 2:**
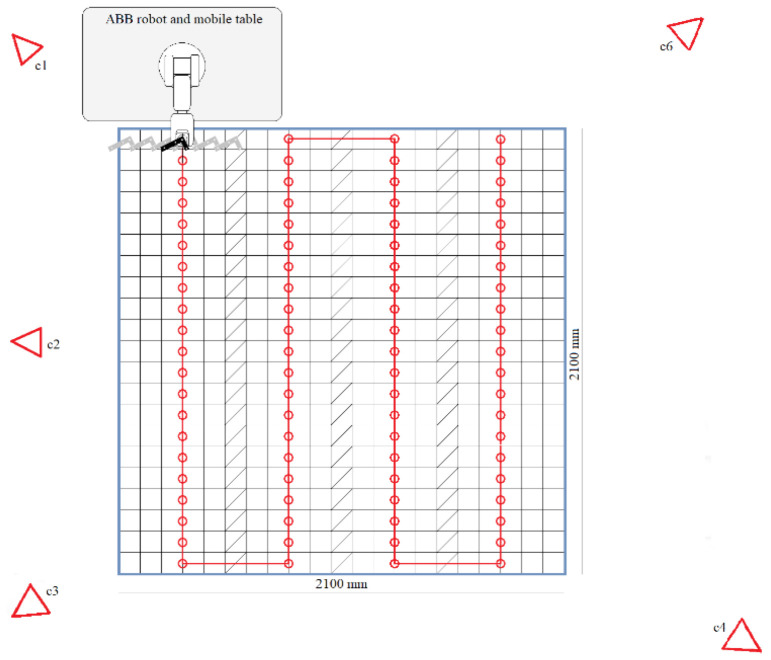
Robot motion test procedure. The red dots are the swept line’s centre, where the tool of the robot is relocated. The 5th camera, C5, is out of the image, 4.4 m in front of camera C2.

The robotic arm was attached to this rigid wheeled table and accurately calibrated following the manufacturer’s guidelines [27]. The arm could perform movements with a maximum horizontal range of 580 mm and position repeatability of 0.01 mm. The robot was programmed to make a horizontal line composed of 5 10-cm-long segments. This was made by travelling the 50 cm line, with 6 stop points. The line was repeated *n* = 3 times before manually moving the robot and the wheeled table to the next zone to be evaluated, following the locations (red dots) in Figure 2. For each movement of the table, the separation between each pair of lines drawn by the robot was 10 cm, and the stops at the ends of the lines overlapped with those of adjacent lines in the same row of the grid.

The robot’s motion verified that:

- The distance between each stopping point and the next (segment length) was 10 cm, with a rotation between them of 0 rad.
- At each stop the robotic arm remained stationary, so the position and rotation should not vary.
- For each repetition of the same stop, the stopping points were in the same coordinates (with a 0.01 mm margin).
- The set of all of the stopping points was in the same plane, and each line consisted of five segments with the same slope.

### 2.2. Data Analysis

**Figure 3:**
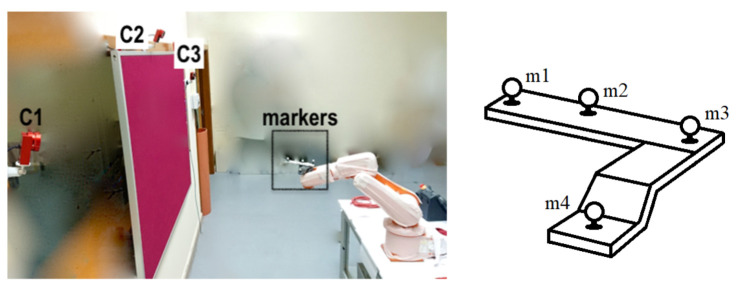
The experimental evaluation procedure: an L-shaped set of markers are moved by the robot in the workspace.

Data were collected by pooling the cameras and then post-processed, including 3D reconstruction, trajectory smoothing, gap filling, and data export to a .csv file at 100 Hz using the manufacturer’s software (Motive: Tracker 2.1.1. Final). The 3D pose of the rigid body, position, and orientation was estimated via the detection of four markers placed at the end of the robotic arm (Figure 3), and the coordinates were referred to the base of the measurement volume established in the calibration phase. The manufacturer recommends placing the retro-reflective markers in a distinctive arrangement that allows the software to more clearly identify the markers on each rigid body during capture. With an L-shape, markers m1 and m3 were at a distance of 16.28 cm. Marker m2 was 6.8 cm away from marker m1 and slightly distanced from the line that would join markers m1 and m3. Marker m4 was 8.8 cm away from marker m3 and 3.6 cm lower than the other 3. The centre of mass of the marker set (calculated by the camera software) was considered to be the centre of the robot tool, and was the point of reference for the calculation of the errors.

A total of *p* = 396 stop points distributed within the large 2D measurement area were collected and set, composed of 66 lines of 5 segments (m = 6 stop points per line). For each point, *n* = 3 repetitions were made, taking K = 60 frames in each. This window was applied in the static part of the movement, once the robotic arm had already stabilized. On this dataset, the mean error of each segment, and the error spread and spatial repeatability at each stop point, were calculated, both in orientation and distances.

The capture area was divided into a grid of 21 × 21 cells of 10 × 10 cm^2^ in size for the study of error spread and spatial repeatability. The programmed movement of the robot together with the displacement of the table covered the entire grid, with at least one stop in each cell. In the case of the mean error, the position of the stops used for the representation was the midpoint of the segment. Because of this, the area was divided into a grid of 20 × 21 cells (one minus in the *x*-axis). The grids were coloured in the form of a heat map.

When data loss occurred at certain stops, the manufacturer’s software autocompleted these locations without information from previous data it was able to measure. These values set by the software were considered to be a limitation of the capture system in the context of this work. Therefore, these autocompleted values were detected and omitted in the error calculation and, to indicate this absence of information, the heat map cell corresponding to the missed stop was left blank. For the calculation of the error spread and spatial repeatability, positions in which at least one repetition experienced this loss of data were discarded. For the mean error, as this was calculated from two stops, positions where one of the two stops experienced losses were discarded.

For each statistic, the errors are summarised as mean, median, and standard deviation, in both the large area and the inner area. Moreover, each heat map is accompanied by the analysis of the overall error distribution in the whole measurement area. Knowledge of the distribution allows us to select a suitable statistical test for the comparison of errors in the two defined areas. In this case, because the results show positive and asymmetric non-Gaussian distributions, a Mann–Whitney U test for comparison of medians was selected [28,29].

#### 2.2.1. Mean Error

**Figure 4:**
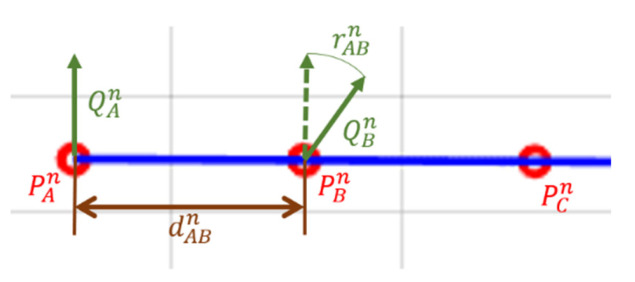
Mean error calculation for distances and orientation. The red circles represent the stops of the robot tool in the same repetition and during the trajectory shown in the image as the blue line.

Mean error is defined as the degree of agreement between the “true” and the experimental values, according to [20] (see Figure 4). In the case of the distance error, it is the difference between the length of the segment travelled by the robot tool and the recorded distance (Equation (1)). Mean error for distances is computed with:

(1) $${\mathrm{Mean}}_{d}\left(P_{A}^{n},P_{B}^{n}\right)=|\;d_{ref}-d_{AB}^{n}\;|=\left|\;d_{ref}-\sqrt{{\left({\bar{x}}_{a}^{n}-{\bar{x}}_{b}^{n}\right)}^{2}+{\left({\bar{y}}_{a}^{n}-{\bar{y}}_{b}^{n}\right)}^{2}}\right|$$

where $Mean_{d}$ is calculated as the Euclidean distance between the recorded segment end positions $P_{A}^{n}\;\mathrm{and}\;P_{B}^{n}$ at repetition *n*. $P_{A}^{n}\;\mathrm{and}\;P_{B}^{n}$ are defined by the pairs of coordinates ${(\bar{x}}_{a}^{n},\;{\bar{x}}_{b}^{n})\;\mathrm{and}\;\left(\;{\bar{y}}_{a}^{n},{\bar{y}}_{b}^{n}\right)$, respectively. ${\bar{x}}_{a}^{n},\;{\bar{x}}_{b}^{n},\;{\bar{y}}_{a}^{n},\mathrm{and}\;{\bar{y}}_{b}^{n}$ are the averaged collected coordinates at these points. The value $d_{ref}$ represents the real distance travelled by the robot tool—in this case, equal to 10 cm—while $d_{AB}^{n}$ is the recorded distance. Points $P_{A}^{n}$ and $P_{B}^{n}$ are referenced to the position of the centre of mass of the marker set.

The orientation error $Mean_{o}$ is the deviation in the orientation of the tool between the two ends of the segment (Equation (2)), characterized as the angular distance between the segment end orientations (orientation of the rigid body attached in the robot tool) expressed as unit quaternions ($Q_{A}^{n},\;Q_{B}^{n}$) for repetition *n*. This angular distance metric is defined as the short path, a geodesic, on the unit sphere ($S^{3}$) [30]. Then:

(2) $${\mathrm{Mean}}_{o}\left(Q_{A}^{n},Q_{B}^{n}\right)=\left|r_{ref}-r_{AB}^{n}\right|=\left|2\cos^{-1}\left(\left|{\bar{q}}_{A}^{n}\;\cdot \;{\bar{q}}_{B}^{n}\right|\right)\right|$$

where ⋅ denotes the inner product of the vectors. The range of values mapped by ${\mathrm{Mean}}_{o}$ is [0,π] (radians). ${\bar{q}}_{a}^{n},\;\mathrm{and}\;{\bar{q}}_{b}^{n}$ are the average collected orientation at these locations. The value $r_{ref}$ represents the real rotation experienced by the robot tool—in this case, equal to 0 rad—while $r_{AB}^{n}$ is the recorded rotation.

For the six stops on each line, a total of five average errors were calculated—one for each segment. The results of the three repetitions per segment were averaged for representation in the heat map. The position of the stops used for the representation is the average of the midpoints of the segment.

#### 2.2.2. Error Spread

Error spread value includes the standard deviation of error at a stop point for a repetition. For the case of the error spread in the distance $Spread_{d}$, this is calculated from the Euclidean distance of the samples to the mean of the data. Then:

(3) $$Spread_{d}\left(P^{n}\right)=\sqrt{\frac{1}{K-1}\overset{K}{\underset{k=1}{\sum }}\left({\left(x_{k}^{n}-{\bar{x}}^{n}\right)}^{2}+{\left(y_{k}^{n}-{\bar{y}}^{n}\right)}^{2}\right)}$$

where $Spread_{d}$ is the error spread for position $P^{n}\;$ values at repetition *n*, while $x_{k}^{n}\;\mathrm{and}\;y_{k}^{n}$ are the coordinates in $P^{n}$ at frame k.

The orientation statistic $Spread_{o}$ is calculated from the angular distance of the rotations collected as quaternions and the mean rotation of the set. Then:

(4) $$Spread_{o}\left(Q^{n}\right)=\sqrt{\frac{1}{K-1}\overset{K}{\underset{k=1}{\sum }}{\left(2\cos^{-1}\left(\left|q_{k}^{n}\;\cdot \;{\bar{q}}^{n}\right|\right)\right)}^{2}}$$

where $Spread_{o}$ is the error spread for rotation $Q^{n}$ values at repetition *n*, and $q_{k}^{n}$ is the quaternion in this pose.

For the six stops on each line, a total of six average errors were calculated—one for each point. The results of the three repetitions per point were averaged for representation in the heat map. The position of the stops used for the representation is the average of the recorded positions of the three repetitions.

#### 2.2.3. Spatial Repeatability

**Figure 5:**
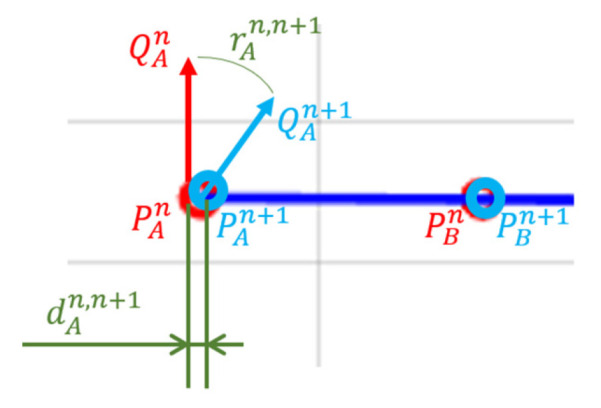
Spatial repeatability calculation for distances and orientation. The red and blue circles represent the stops of the robot tool in two different repetitions and during the trajectory shown in the image as the blue line.

Lastly, spatial repeatability is calculated as the error composed by small variations in the pose between successive observations taken under identical conditions (see Figure 5). In the case of distances, ${\mathrm{Rep}}_{d}$ is calculated as the difference between the measurements of the same point in two successive repetitions $P^{n}\;\mathrm{and}\;P^{n+1}$. Then:

(5) $${\mathrm{Rep}}_{d}\left(P^{n},P^{n+1}\right)=\sqrt{{\left({\bar{x}}^{n}-{\bar{x}}^{n+1}\right)}^{2}+{\left(\;{\bar{y}}^{n}-{\bar{y}}^{n+1}\right)}^{2}}$$

where ${\bar{x}}^{n},\;{\bar{x}}^{n+1},{\bar{y}}^{n},\mathrm{and}\;{\bar{y}}^{n+1}$ are the averaged collected coordinates at these points.

For orientations, $Rep_{o}$ is the angular difference extracted between $Q^{n}\;\mathrm{and}\;Q^{n+1}$. Then:

(6) $$Rep_{o}\left(Q^{n},\;Q^{n+1}\right)=\left|2\cos^{-1}\left(\left|{\bar{q}}^{n}\;\cdot \;{\bar{q}}^{n+1}\right|\right)\;\right|$$

where ${\bar{q}}^{n}\;\mathrm{and}\;{\bar{q}}^{n+1}$ are the averaged quaternions at these points.

For the three repetitions of each line, a total of two average error lines were calculated, with six stops on each one. The results of the two errors per point were averaged for representation in the heat map. The position of the stops used for the representation is the average of the recorded positions of the three repetitions.

#### 2.2.4. Experimental Inner Areas

For each statistic described, the internal area with the smallest error was studied, comparing it with the internal area recommended by the manufacturer after the calibration process. For this purpose, the measurement area was divided into all possible 9 × 9 cell windows, and those in which at least one cell experienced losses and did not contain an error value were discarded. This process was performed twice—once for the 21 × 21 cell configuration, and once for the 20 × 21 cell configuration. The errors of each statistic that fell within each of the windows were averaged and compared by selecting the lowest values.

## 3. Results

### 3.1. Calibration and Data Collection

For the OMC calibration procedure, the number of frames used was at the top level of the manufacturer’s recommendations (more than 5000 frames). The nominal residual error achieved was 1.1276 mm (mean) with the 99th percentile of 3.505 mm.

**Figure 6:**
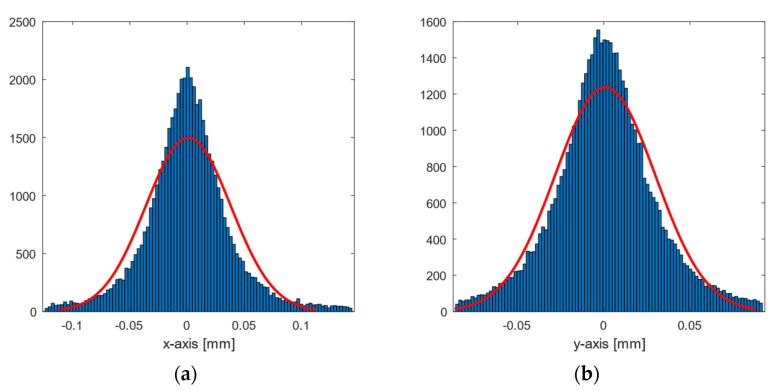
Histogram plot of the 97th percentile for inner area data error concerning their means on the (**a**) *x*-axis and (**b**) *y*-axis. The red line represents a normal distribution as a comparison.

Figure 6 plots the error (97th percentile) of the inner area measurements around their mean on both axes. The error distribution is presented as a bell-shaped distribution with dispersions of 0.06 and 0.05 mm on the *x*-axis and *y*-axis, respectively. By comparison, a 1 s measurement (1000 samples) was taken of the rigid body at rest at a position located within the inner area. These data recorded a dispersion of 0.02 mm on both axes, 0.03–0.04 mm less than in the measurements of the experiment.

**Figure 7:**
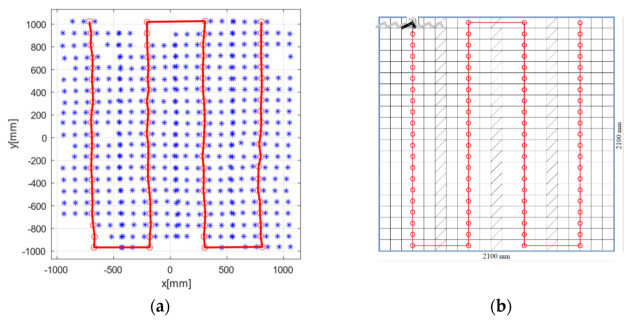
(**a**) Measured robot motion, with the stop positions in blue. The red dots are the sweep line’s centre, where the robot is relocated. (**b**) Robot motion test procedure.

Figure 7 shows the results of the robot motion test procedure and the estimate of the centre of the sweep line. Data loss occurred at certain stops, mainly at the outer limits, and these positions were discarded. Because of this, the perimeter of the area in the heat maps became irregular.

The data losses during the measurements were consistent with the expected margins, with it being possible to calculate the errors in all of the cells in the inner area, while in the outer area it was impossible to calculate 46 of 441 cells (56 of 420 for mean error stops).

**Figure 8:**
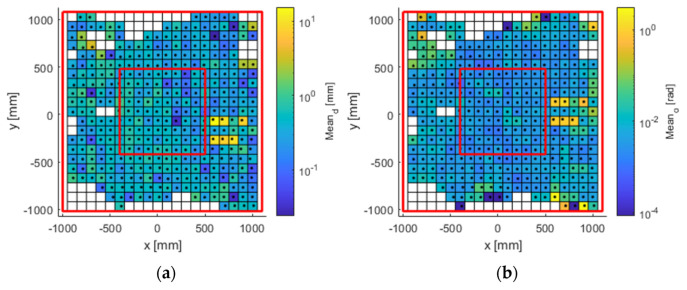
Mean error in (**a**) position (mm); and (**b**) orientation (rad). The available work area. The red lines delimit the large and inner areas.

**Figure 9:**
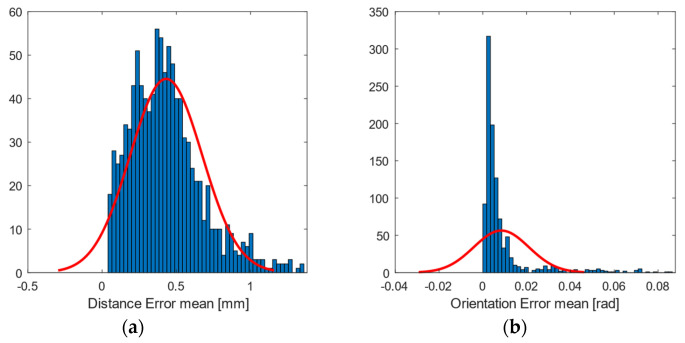
Histogram plot of the 97th percentile for mean error in (**a**) position (mm); and (**b**) orientation (rad). The red line represents a normal distribution as a comparison.

**Figure 10:**
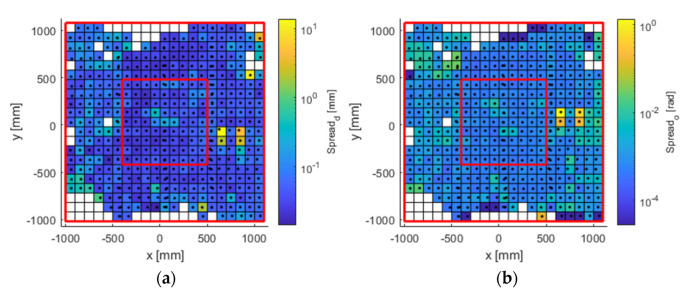
Error spread in (**a**) position (mm); and (**b**) orientation (rad). The available work area. The red lines delimit the large and inner areas.

**Figure 11:**
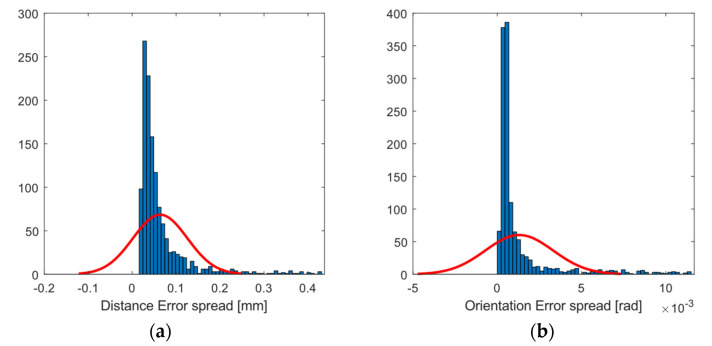
Histogram plot of the 97th percentile for error spread in (**a**) position (mm); and (**b**) orientation (rad). The red line represents a normal distribution as a comparison.

**Figure 12:**
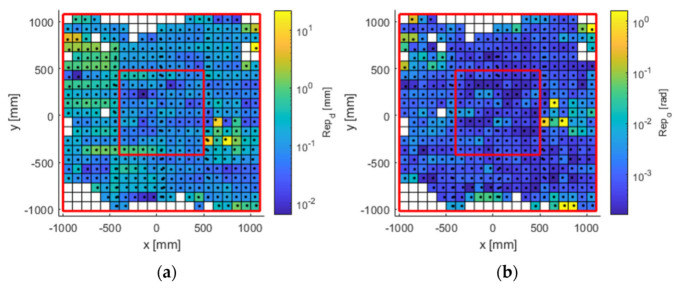
Spatial repeatability error in (**a**) position (mm); and (**b**) orientation (rad). The available work area. The red lines delimit the large and inner areas.

**Figure 13:**
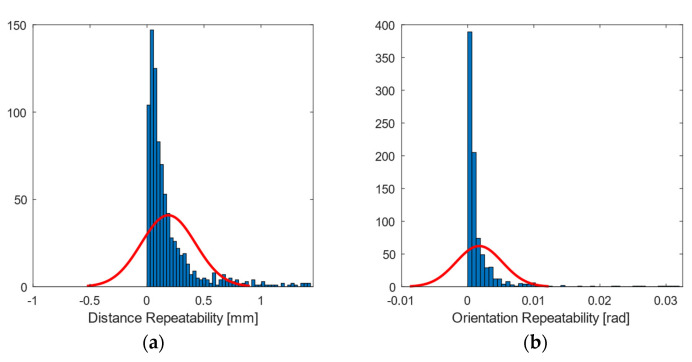
Histogram plot of the 97th percentile for spatial repeatability in (**a**) position (mm); and (**b**) orientation (rad). The red line represents a normal distribution as a comparison.

The experimental results obtained for the entire workspace represented in Figure 1 are presented below. Figures 8, 10, and 12 represent successively, in the form of a heat map with a topmost view, the results at each point in the workspace for the six defined uncertainty metrics. A logarithmic colour scale is used. Figures 9, 11, and 13 show the distribution of the complete area for each error, together with a Gaussian distribution of equal mean and dispersion as a comparison.

### 3.2. Mean Error

Mean distance error (Figure 8 and Figure 9) was generally below 1 mm (green tone) in all registered positions. The accuracy errors in orientation were below 1° deg (approx. 0.02 rad, blue tones), and were distributed in zones similar to the error of position. In the case of the heat maps for the mean error, there was no clear visual improvement between the outdoor and indoor samples.

### 3.3. Error Spread

Distance error spreads (Figure 10 and Figure 11) fluctuated below hundredths of 1 mm over much of the working area. Error spread tends to grow on the sides, especially on the left side of the area. Orientation errors show a similar topological distribution, with values below 0.14° (approximately 0.003 rad, blue tones).

### 3.4. Spatial Repeatability Error

Spatial repeatability error (Figure 12 and Figure 13) had values below 0.5 mm (blue tone) in central areas. The zoning as a function of the position of the cameras showed a higher error in the lateral areas. As for the orientation, it had values below 0.3 deg (approx. 0.005 rad), rising to an order of magnitude on the sides, as in the previous error metrics referring to orientation.

### 3.5. Mean Error in the Inner and Large Work Areas

As the error distribution was found to have an asymmetric bell-shaped distribution, it was necessary to use another statistic than the mean or standard deviation for the definition of the error in the areas. Table 1 shows the mean value, standard deviation, and median of each distance error metric by averaging all the values. The calculation was made for both reference areas—the inner work area in Figure 1, and the large area—excluding stop points that were in the inner area.

The means and variances for all of the metrics improved in the inner area, as expected, especially for distance accuracy. The same is true for the calculation of the median statistics, except for the case where the mean distance error has a lower median in the large area than in the inner area. Table 2 shows similar results, but refers to orientation.

The errors have positive and asymmetric non-Gaussian distributions. A Mann–Whitney U test was performed in order to validate the null hypothesis that the medians of the errors in both areas are equal—or, on the contrary, significantly different. The results are shown in Table 3.

With a criterion of *p* > 0.05 to reject the null hypothesis, the results show that the calculated errors are not significantly different in the median in the case of the mean distance error. For all other errors, we can reject the null hypothesis and consider errors in the two areas to be significantly different.

### 3.6. Experimental Inner Areas

**Figure 14:**
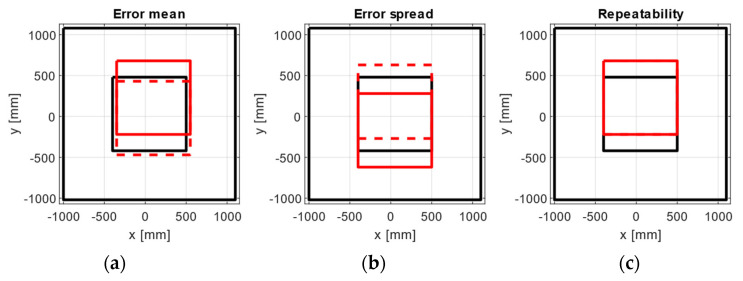
Inner areas with the lowest errors (red lines) for (**a**) mean error, (**b**) error spread, and (**c**) repeatability in positions (solid lines) and orientations (dashed lines). The black squares represent the inner and large areas.

For each metric analysed, the area of 0.9 × 0.9 m^2^ with the smallest cumulative value was calculated (see Figure 14). The areas for all metrics were located close together on the *x*-axis, near to the one established with the manufacturer’s software.

Table 4 shows the mean, standard deviation, and median values for the newly calculated inner areas. The values of the three statistics are lower than those shown in Table 1 and Table 2.

## 4. Discussion

The proposed methodology for the evaluation of the optical system allows an exhaustive analysis of the distributed error in the capture area, with the estimation of the statistical parameters of mean error, error spread, and spatial repeatability—a metric not available with manual evaluation processes, and useful in applications requiring precision and deterministic systems. The area in which this was applied was a horizontal plane of greater extension than the reach of the robotic arm, and which was significantly enlarged compared to that recommended by the manufacturer, forcing the failure. This experiment was designed to study the capacity of the evaluation method to detect the limitations of the capture system.

The extension of the experiment to a 3D environment could have been achieved by repeating the experimental procedure with the robot mounted at different heights, but the analysis of each plane would have been the same as the one performed for the mid-height plane we used, and the calculation of the error distribution on the vertical axis was the same as on the horizontal one. For this reason, we decided to simplify the experiments to a single horizontal plane in order to facilitate the analysis and compression of the results.

The analysis of the dependence of the error on the number of cameras was also considered. However, as with the 3D study, varying the number of cameras would not have changed the way the statistics were applied for each configuration, but it would have increased the complexity of the results.

Calibration of the OMC laboratory following the manufacturer’s instructions produced a nominal position error of 1.1276 mm (mean) at the 99th percentile of 3.505 mm (values given by the manufacturer software as result of calibration). For robotic applications, the manufacturer recommends an equidistant distribution of the cameras around the setup, separating them by a consistent distance. However, in most cases, this ideal configuration cannot be applied. For these cases, as in [1], we chose to make a placement adapted to our laboratory, with an irregular distribution of the cameras, like the kind we can find in a common laboratory session. Despite this, in our systematic evaluation of the entire workspace, a lower value (0.6239 mm) of position error was obtained, but not considering the samples that could not be reconstructed by the manufacturer’s software. The methodology used allows for a more detailed study.

The visualisation of heat map errors provides an insight into the strong relationship between performance and the selected configuration of the capture system. A mere visual inspection of Figure 8, Figure 9, Figure 10, Figure 11, Figure 12 and Figure 13 reveals a decrease in all errors in the interior area, as is reflected in Table 1 and Table 2. Some positions located in the large area, mainly at the outer limits, were affected by the reduction in the number of cameras capable of viewing this area. 3D reconstruction was directly affected, and data loss occurred at certain stops (see Figure 7). The loss of accuracy at the edges has already been highlighted before [1], with magnitude compatible to that reported here.

Another feature of the capture system configuration seen in the heat maps is that the first third of the work area, starting from the left, generally has higher error than the other two thirds. This increase in error and data loss is due to characteristics of our setup, which is too close to the line of cameras C1–C2–C3. As camera C6 is further away from the area, its field of view covers a larger area than camera C2.

The mean positional error was reduced from 0.6999 to 0.4389 mm in the inner area (Table 1), as was the case with the value of its dispersion. However, the distribution of these errors appears as an asymmetric and positive bell-shaped distribution with a higher median in the inner area than in the outer area. A Mann–Whitney U test was carried out in order to check the similarity between the two median values, and the results showed that, in terms of mean distance error, we cannot conclude that they are significantly different in both areas. For the dispersion and repeatability metrics for both distances and orientations, the values decreased in the inner area. This difference was supported by the Mann–Whitney U test, which showed that we can reject the null hypothesis and consider errors in the two areas to be significantly different.

Spatial repeatability errors in distance and orientation (Figure 12) were of special interest, since they could not be studied in other similar works. This was made possible by using an industrial robot, which allowed the movements to be repeated with great precision without human intervention. Repeatability errors showed values higher than the error given by the manufacturer (0.01 mm). This may be due to the sensitivity in the 3D reconstruction, which when receiving a small variation in the position of the markers, translates into a greater displacement of the rigid body [31]. Another possibility is the existence of dependence between the current sample and the previous ones, meaning that two sets of markers located in the same spatial positions could be wrongly interpreted as different body poses.

**Figure 15:**
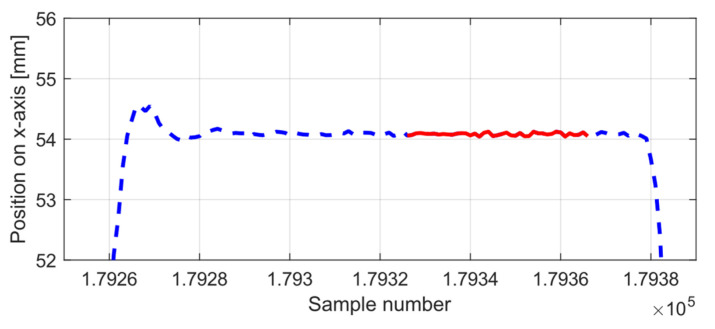
Example of selecting the *x*-axis stop data (solid red line) from the raw robot motion data (dashed blue line).

On the other hand, placing the robot on a moving table that uses a braking mechanism to stop it may raise the question of whether this will be enough to prevent the movement of the cart. The data window corresponding to each stop of the robot was manually selected by discarding the vibrations produced by the robot during movement, as shown in Figure 15. Despite this, the possible error introduced was examined by analysing the standard deviation of the measurements around its mean (Figure 6) for samples from the inner area, and for a one-second sample in which the rigid body was completely at rest. The dispersion of the experiment shows a value between 0.03 and 0.04 mm higher than the value at rest, so we consider that these variations can be disregarded as insignificant.

The results obtained (mainly for the mean error and error dispersion) were close to those reported in other methodologies with manual procedures [1,2,18], and they confirm the possibility of using an industrial robot as an alternative for the evaluation of an optical system, which facilitates this process by not requiring the acquisition of extra tools. In this type of cell, where the quality of measurements in the robot’s area of motion may be of primary interest, its use could lead to the automation of the calibration and evaluation process.

### 4.1. Capture System Useful Area

The evaluated capture area consisted of a horizontal plane of greater extension than that recommended by the manufacturer. Although with the design of the experiments it was expected to find these errors in the end zones, the useful area for measurement exceeded that recommended by the manufacturer (understood as the area recommended as perceptible by the six cameras). On the other hand, Figure 8, Figure 9, Figure 10, Figure 11, Figure 12 and Figure 13 suggest that the “true centre” of our OMC was somewhat shifted to the right of the centre indicated by the manufacturer in the workspace. Irregular positioning of the cameras caused a higher loss of measurements on the left side of the capture area. This implies that when wanting to use the largest possible measurement area, the centre of the measurement area will be shifted to the right, or, on the other hand, that the cameras should be repositioned in such a way that camera C2 is moved away from the centre, in order to increase its visibility of the measurement area.

To propose an experimental central area of the same size as the evaluated inner area, based on the statistics studied (mean error, error spread, and repeatability), the internal area of 0.9 × 0.9 m^2^ containing the smallest error for each statistic was calculated (see Figure 14). The results are consistent with the location of the error exhibited by the heat maps, with all experimental areas lying in the same vertical strip and located within the overlapping capture volume of the six cameras.

### 4.2. Applicability of the Methodology

This experiment was designed to study the capacity of the evaluation method to detect the limitations of the capture system. For this purpose, the capture volume was simplified to a horizontal plane, and was tested with an irregular and fixed number of cameras in a larger area than that recommended by the manufacturer. However, depending on the application, the size, shape and configuration of the capture volume may vary. The proposed evaluation can be adapted to other robot paths as long as the facts remain the same:

- The route is planned as a set of segments of known size, independent of the direction of the movement.
- At the end of each segment, the robot remains completely stationary, so the position and rotation should not vary.
- Each segment must be repeated a minimum of two times in order to apply the spatial repeatability statistic.

## 5. Conclusions

When OMC systems are applied to monitoring human–robot collaborative cells, the evaluation of the metrology of the optical system often requires procedures with custom-made calibrated devices. In collaborative environments, available industrial robots can be used to robotize this task and expedite the use of these facilities. This work details the results of a methodology to evaluate the metrological performance of OMC spaces dedicated to human–robot collaborative environments, taking advantage of the performance of an industrial robot. The methodology was based on the ASTM E3064 test method, but it was implemented with the existing robot in the cell. The results obtained allow the detailed analysis of the error distribution, and allow the characterisation of the area to be evaluated and the metrological characteristics to be obtained in a way consistent with other methods based on human intervention. The use of an industrial robot in this context would allow the automation of the process of calibration and evaluation of the robot in its working area.

## Figures and Tables

**Table 1:** Mean, standard deviation, and median of distance error metrics.

	Mean Error			Error Spread			Repeatability		
	Mean	Std	Median	Mean	Std	Median	Mean	Std	Median
Inner area	0.4257	0.2069	0.4382	0.0523	0.0547	0.0369	0.1369	0.1674	0.0865
Large area	0.6852	1.5407	0.3982	0.1866	0.9922	0.0482	0.4702	1.9574	0.1391
All	0.6239	1.3541	0.4088	0.1599	0.8899	0.0442	0.4040	1.7583	0.1264

**Table 2:** Mean, standard deviation, and median of orientation error metrics.

	Mean Error			Error Spread			Repeatability		
	Mean	Std	Median	Mean	Std	Median	Mean	Std	Median
Inner area	0.0040	0.0033	0.0031	0.0009	0.0013	0.0005	0.0009	0.0015	0.0005
Large area	0.0617	0.2983	0.0054	0.0088	0.0780	0.0007	0.0298	0.1973	0.0009
All	0.0481	0.2618	0.0042	0.0072	0.0699	0.0006	0.0241	0.1769	0.0008

**Table 3:** Mann-Whitney *U*-test results.

	*p*-Values		
	Mean Error	Error Spread	Repeatability
Distance	0.59	1.82 × 10^−9^	4.71 × 10^−5^
Orientation	1.57 × 10^−17^	1.59 × 10^−8^	1.31 × 10^−9^

**Table 4:** Mean, standard deviation, and median for experimental inner areas.

	Mean Error			Error Spread			Repeatability		
	Mean	Std	Median	Mean	Std	Median	Mean	Std	Median
Distance	0.3979	0.1861	0.4019	0.0481	0.0530	0.0349	0.1003	0.0792	0.0824
Orientation	0.0039	0.0031	0.0031	0.0008	0.0013	0.0004	0.0009	0.0014	0.0004

## Data Availability

The data presented in this study are available on request from the corresponding authors.
